# A polymorphism in porcine miR-22 is associated with pork color

**DOI:** 10.3389/fvets.2022.939440

**Published:** 2022-07-28

**Authors:** Han Wang, Zhonghao Shen, Ruihua Huang, Ayong Zhao, Jiani Jiang, Pinghua Li, Xiaolong Zhou, Songbai Yang, Liming Hou

**Affiliations:** ^1^Key Laboratory of Applied Technology on Green-Eco-Healthy Animal Husbandry of Zhejiang Province, Department of Animal Breeding, College of Animal Science and Technology, College of Veterinary Medicine, Zhejiang A&F University, Hangzhou, China; ^2^Institute of Swine Science, Department of Animal Breeding, College of Animal Science and Technology, Nanjing Agricultural University, Nanjing, China; ^3^Department of Statistical Sciences, University of Toronto, Toronto, ON, Canada

**Keywords:** pig, meat color, miR-22, *ELOVL6*, intracellular calcium concentration, mutation

## Abstract

MicroRNAs (miRNAs) are posttranscriptional regulators that play key roles in meat color regulation. Changes in miRNA expression affect their target mRNAs, leading to multifunctional effects on biological processes and phenotypes. In this study, a G > A mutation site located upstream of the precursor miR-22 sequence in Suhuai pigs was significantly correlated with the meat color parameter a^*^(redness) of the porcine longissimus dorsi (LD) muscle. AA genotype individuals had the highest average meat color a^*^ value and the lowest miR-22 level. When G > A mutation was performed in the miR-22 overexpression vector, miR-22 expression significantly decreased. Considering that Ca^2+^ homeostasis is closely related to pig meat color, our results further demonstrated that *ELOVL6* is a direct target of miR-22 in pigs. The effects of miR-22 on skeletal muscle intracellular Ca^2+^ were partially caused by the suppression of *ELOVL6* expression.

## Introduction

MicroRNAs (miRNAs) are posttranscriptional regulators that play key roles in meat color regulation (1). Among these, meat color has a major impact on consumer preference and market price (2). Pig meat color is a complex quantitative trait with low heritability, ranging from 0.14 to 0.25 (3). The International Commission on Illumination (CIE) defined a colorimetric system that is widely used in meat color detection (4). According to CIE, any kind of object color characteristics can be represented by tri-stimulus values (i.e., X, Y, and Z). Through mathematical relationship conversion, the colorimeter converts the original CIE tri-stimulus values into understandable values, such as L^*^ (lightness), a^*^(redness), and b^*^(yellowness). Because meat color can only be obtained after slaughter, early breeding for meat color is difficult. Recent advances in porcine genomics studies have applied whole-genome sequencing (WGS) and genome-wide association study (GWAS) to improve our understanding of the genetic regulation of pork meat quality by identifying quantitative trait loci (QTLs), candidate genes, and related genetic variants associated with pork meat quality traits (5). Thus, new tools are being developed to improve pig meat color traits using marker-associated selection (MAS) and genome selection (GS) approaches.

MicroRNAs are posttranscriptional regulators that play key roles in meat color regulation. Genetic variation in miRNA genes can alter precursor miRNA (pre-miRNA) transcription, affecting the processing or stability of pre-miRNA and the expression of mature miRNAs. These changes in turn affect target mRNAs, leading to multifunctional effects on individual phenotypes (6, 7). Genetic mutations in miR-208b and miR-1 precursor genes are significantly associated with pig muscle fiber characteristics and meat color traits (8, 9). The expression of miR-499 is significantly correlated with the expression of *myoglobin*, which typically reflects meat color (10). However, few studies have explored the mechanism of miRNA involvement in the regulation of pig meat color traits.

MiR-22 is a miRNA that plays key roles in multiple biological processes, including tumor suppression, cancer therapy, and the prevention of cardiac hypertrophy (11). Previous studies have shown that miR-22 inhibits the proliferation of porcine muscle satellite cells (PMSCs) and promotes their differentiation (12). MiR-22 is also involved in the regulation of muscle fiber type conversion *via* the inhibition of the *AMPK*-*SIRT1*-*PGC-1*α pathway in mouse muscle cells (13). Muscle fiber type is associated with meat color (14), and previous studies have demonstrated that Ca^2+^ homeostasis affects pig meat color (15, 16).

Through bioinformatics prediction, we discovered that elongase of very-long-chain fatty acids 6 (*ELOVL6*), an elongase that catalyzes *de novo* synthesis of fatty acids (17), is a direct potential target gene of miR-22. In our previous study, we showed that *ELOVL6* is more highly expressed in white longissimus dorsi (LD) muscle than in red soleus (SOL) muscle (18). In *ELOVL6*-knockout *Drosophila*, the proportion of stearic acid in mitochondria was repressed. However, when stearic acid was added to food consumed by *Drosophila*, stearoylation was promoted, which inhibited the *JNK* signaling pathway and reduced the ubiquitination of mitochondrial fusion proteins, thereby promoting mitochondrial metabolism and fusion and maintaining its normal function (19). Dysfunction due to the inhibition of mitochondrial fusion decreased the sensitivity of myofibroblasts to Ca^2+^ action (20). The *ELOVL6* gene may affect the concentration of Ca^2+^ in muscle cells, influencing pig meat color. However, few studies have investigated the involvement of *ELOVL6* in muscle cell Ca^2+^ concentration.

Therefore, we hypothesized the existence of a functional mutation site related to meat color in the precursor sequence gene of porcine miR-22, which regulates Ca^2+^ concentration in porcine muscle cells by affecting the expression of miR-22. In this study, we explored the role of miR-22 in regulating skeletal muscle intracellular Ca^2+^ by investigating a mutation site related to pork color in the miR-22 gene. Our findings will provide a useful theoretical basis for future research on genetic markers of pig meat color traits.

## Materials and methods

### Animals and phenotype measurements

All animal procedures were performed in accordance with the Guidelines for Care and Use of Laboratory Animals of Nanjing Agriculture University and approved by the Animal Ethics Committee of Nanjing Agriculture University. A total of 300 healthy Suhuai pigs (i.e., 194 castrated barrows and 106 sows) with the same market weight (80–90 kg) and approximately 7–8 months old were reared at the Huaiyin Breeding Farm (Huaian, China) under the same feeding conditions and slaughtered at Sushi Meat Products Co., Ltd. (Huaian, China).

Ear tissues from the end of the right ear were collected and stored in 75% alcohol for DNA extraction. Samples of the LD, SOL, psoas major (PM), masseter (MA), and biceps femoris (BF) muscles were collected for RNA extraction from four randomly selected healthy Suhuai pigs with similar body weight and age. LD muscle samples from the last rib of the carcass were collected and used to determine meat color. In China, traditional hot fresh meat is usually marketed immediately after slaughter, whereas chilled fresh meat is often cooled quickly to a stable 0–4°C within 24 h postmortem and maintained at this temperature until sold to the consumer (21). Therefore, meat color (L^*^a^*^and b^*^) was measured on the last rib at 2 (room temperature) and 24 h (4°C) postmortem using a chromameter (Minolta Camera Co., Osaka, Japan).

### Cell culture

Porcine muscle satellite cells were isolated as described previously (22). LD muscles of 3-day-old healthy male piglets were disinfected with alcohol and aseptically dissected *in vitro*. Muscle samples were washed three times with phosphate-buffered saline (PBS) (HyClone, Logan, USA) containing 1% penicillin–streptomycin (Solarbio, Beijing, China), and the skin, fat, and connective tissue were removed. The muscle was crushed into meat paste and digested with 2 ml PBS containing 0.1% collagenase II at 37°C for 10 min. The samples were centrifuged three times at 500 rpm for 5 min each. The cell suspension was sequentially passed through 100 and 40 μm nylon cell filters and a 20 μm mesh filter. The filtrate was collected into a 15 ml centrifuge tube and centrifuged at 1,500 rpm for 10 min, and the supernatant was discarded to obtain the cell pellets. The pellets were washed three times with PBS, added to a Percoll gradient solution, and centrifuged at 2,000 rpm for 1 h. Totally, 26% of the Percoll gradient solution was recovered, which then was cultured in growth medium (GM) and Dulbecco's modified Eagle medium (DMEM; HyClone, Logan, USA) supplemented with 10% fetal bovine serum (FBS) and 1% penicillin–streptomycin (Solarbio, Beijing, China). HEK293T cells and PK15 cells were cultured in 12-well plates in DMEM with 10% FBS and penicillin–streptomycin (50 mg/mL). All cells were incubated at 37°C under 5% CO_2_.

### Isolation of genomic DNA and single-nucleotide polymorphism genotyping

Genomic DNA was isolated from ear tissue using the standard phenol–chloroform protocol method (23). DNA concentration and integrity were measured using a Nanodrop 2000 spectrophotometer (Thermo Fisher Scientific Inc., Waltham, MA, USA). A 688 bp DNA fragment encompassing porcine pre-miR-22 and its flanking sequences was amplified by polymerase chain reaction (PCR) using pooled genomic DNA isolated from eight randomly selected pigs. The primer sequences (miR-22-SNP) are provided in Table 1. Polymorphisms were sequenced by TsingKe Inc. and analyzed using the Chromas software. The NC_010454.4:g.47913559 G > A SNP was selected for genotyping and named according to sequence variation nomenclature guidelines provided by the Human Genome Variation Society. Sequences for the 300 pigs were individually amplified and genotyped by PCR using the miR-22-SNP primers (Table 1).

**Table 1:** The primers used for PCR in this study.

**Name**	**Sequence (5^**′**^-3^**′**^)**	**Accession number**
miR-22	F: CAGGAAGCTGCCAGTTGAA R: TCAACTGGTGTCGTGGAGTC	NR_038580.1
RT-loop-miR-22	CTCAACTGGTGTCGTGGAGTCGGCAATTCAGTTGAGACAGTTC	NR_038580.1
*U6*	F: GCTTCGGCAGCACATATACT R: TTCACGAATTTGCGTGTCA	XM_003357006.5
miR-22-SNP	F: GGTCCACATGCTCACCTA R: CGCACGAGGACCAACTAA	N/A
*GAPDH*	F: GATGGTGAAGGTCGGAGTG R: CCAAGTTGTCATGGATGACC	NM_001206359.1
*Myhc I*	F: CGACACACCTGTTGAGAAG R: AGATGCGGATGCCCTCCA	NM_213855.2
*MyhcIIb*	F: GTTCTGAAGAGGGTGGTAC R: AGATGCGGATGCCCTCCA	NM_001123141.1
*Myoglobin*	F: GGATGAGATGAAGGCCTCTG R: AACCTGGATGATGGCTTCTG	NM_214236.1
*ELOVL6*	F: AGCAGTTCAACGAGAACGAAGCC R: TGCCGACCGCCAAAGATAAAG	XM_021100707.1

### Quantitative reverse transcription PCR

TRIzol reagent was used to extract total RNA (24). Briefly, TRIzol (Invitrogen, Waltham, MA, USA) was added to the culture dish to lyse the cells or tissue in the tubes. Then, 0.2 ml chloroform per 1 ml TRIzol reagent was added, and the samples were incubated at 15–30°C for 2–3 min. Then, the samples were centrifuged for 15 min at 12,000× g at 4°C. Following centrifugation, approximately 0.5 ml of the aqueous phase was transferred to a fresh tube, and 0.5 ml of isopropyl alcohol was added. Samples were incubated at 15–30°C for 10 min and centrifuged at 12,000× g for 10 min at 2–8°C. The supernatant was removed, and the RNA pellet was washed once with 75% ethanol and air-dried for 5–10 min. qRT-PCR analyses were performed as described previously (25). Reverse transcription of miRNA was performed using the RR014a reverse transcription kit (Takara, Kusatsu, Japan). Differential expression was analyzed using the 2^−ΔΔCt^ method (26). *U6* and *GAPDH* were selected as housekeeping genes for miR-22 and protein coding genes, respectively. The primer sequences used in these analyses are provided in Table 1.

### Plasmid construction

To construct the pcDNA3.1-miR-22 expression vector, a fragment of the first intron containing the NC_010454.4:g.47913559 G > A SNP and the entire second exon of the miR-22 host gene (TLC-domain containing 2, *TLCD2*) was amplified and cloned into the pcDNA 3.1 plasmid, which was digested with the *EcoRI* and *XhoI* restriction enzymes (Invitrogen). The primer sequences were 5'-GAATTCGGGACCAAGTCAGTTCGG-3′ and 5′-CTCGAGCCAGACTTAGGCAATACAGG-3′. The pcDNA3.1-miR-22 expression point mutant vector was generated using the Mut Express II Fast Mutagenesis kit (Vazyme Biotech Co., Ltd., Nanjing, China). The pcDNA3.1-*ELOVL6* expression vector, *ELOVL6* Psicheck-2 dual-luciferase reporter, and *ELOVL6* point mutant vectors were generated by Tsingke (Hangzhou, China).

### RNA oligonucleotides and transfection

MiR-22 mimics, miR-22 mimics NC, miR-22 inhibitor, miR-22 inhibitor NC, small interfering RNA (siRNA) against pig *ELOVL6*, and the negative control scramble siRNA (NC) were designed and synthesized by RiboBio (Guangzhou, China). The primer sequences are provided in Table 2. The Lipofectamine 3000 system (Invitrogen) was used for transfection according to the manufacturer's instructions.

**Table 2:** Sequence of RNA oligonucleotides.

**Name**	**Forward (5^**′**^-3^**′**^)**	**Reverse (5^**′**^-3^**′**^)**
Negative control	UUCUCCGAACGUGUCACGUdTdT	ACGUGACACGUUCGGAGAAdTdT
miR-22-mimics	AAGCUGCCAGUUGAAGAACUGU	AGUUCUUCAACUGGCAGCUUUU
miR-22-inhibitor	CAGUUCUUCAACUGGCAGCUU	
Inhibitor NC	UCUACUCUUUCUAGGAGGUUGUGA	
siRNA-*ELOVL6*	UGAACAAGCGCGCGAAGUU	AACUUCGCGCGCUUGUUCA
siRNA-Negative control	UUCUCCGAACGUGUCACGU	ACGUGACACGUUCGGAGAA

### Dual-luciferase reporter assay

In 12-well plates, miR-22 or NC mimics (50 nM) were transfected into HEK293T cells with 1 μg Psicheck-2 *ELOVL6* luciferase vector (wild-type or mutated) using Lipofectamine 3000. The assays were performed 24 h after transfection according to the manufacturer's instructions (Promega, Madison, WI, USA).

### Immunofluorescence staining

Porcine muscle satellite cells were cultured in 12-well cell culture plates with cell slides. When cells reached 70–80% confluence, they were washed three times with precooled PBS for 5 min and fixed with 4% paraformaldehyde for 15 min. Furthermore, the cells were permeabilized with 0.25% Triton X-100 for 10 min, blocked at 4°C overnight, and incubated with anti-*Pax7* primary antibody (Abcam, Shanghai, China) or anti-*Desmin* primary antibody (Abcam, Shanghai, China) for 1 h at room temperature. Then, a fluorescent secondary antibody (Thermo Fisher, Shanghai, China) was incubated with the cells for 1 h at room temperature. We added 4′,6-diamidino-2-phenylindole (DAPI) (Invitrogen) to stain the nuclei, and the cells were incubated for 10 min at room temperature. A fluorescence microscope (Olympus, Tokyo, Japan) was used to observe the samples.

### Detection of intracellular calcium concentration

The cells were washed twice with PBS and once with D-Hank's solution. Fura-2/AM (5 μM) was added, and the cells were incubated at 37°C for 30 min. The samples were washed three times and incubated with D-Hank's solution at 37°C for 20–30 min. A fluorescence spectrophotometer with excitation wavelengths of 340 and 380 nm, and an emission wavelength of 510 nm was used to measure R, F_max_, and F_min_, respectively. The following formula was used to calculate intracellular calcium concentration ([Ca^2+^]i): [***Ca***^**2+**^]***i*****=*****Kd***[***R*** **−** ***Rmin***)/(***Rmax*** **−** ***R***)](***Fmin*****/*****Fmax***), where Kd = 224 nmol/L, R is the ratio of fluorescence intensity measured at excitation wavelengths of 340/380 nm, R_max_ is the same ratio after adding Triton X-100, and R_min_ is the same ratio after adding ethylene glycol tetraacetic acid (EGTA). F_max_ and F_min_ are the maximum and minimum fluorescence intensities measured at 340 nm after adding Triton X-100 and EGTA, respectively.

### Statistical analysis

Association analysis of the SNP for meat color was performed using the PROC GLM procedure in the SAS v9.2 software (SAS Institute Inc., Cary, NC, USA), with both sex and SNP as fixed effects and slaughter age as a covariate (27, 28). Kinship was not taken into account in this statistical model. The associated genotype mean eigenvalues were compared using the Tukey–Kramer program in SAS to detect significant differences. All data were expressed as mean ± standard error of the mean (SEM). Unpaired Student's *t*-tests were used to calculate *P-*values using the SPSS v20.0 software (SPSS Inc., Chicago, IL, USA). Significant differences were evaluated at a level of *P* < 0.05 and highly significant differences at *P* < 0.01.

## Results

### Expression profiling of miR-22 in different porcine muscle types

**Figure 1:**
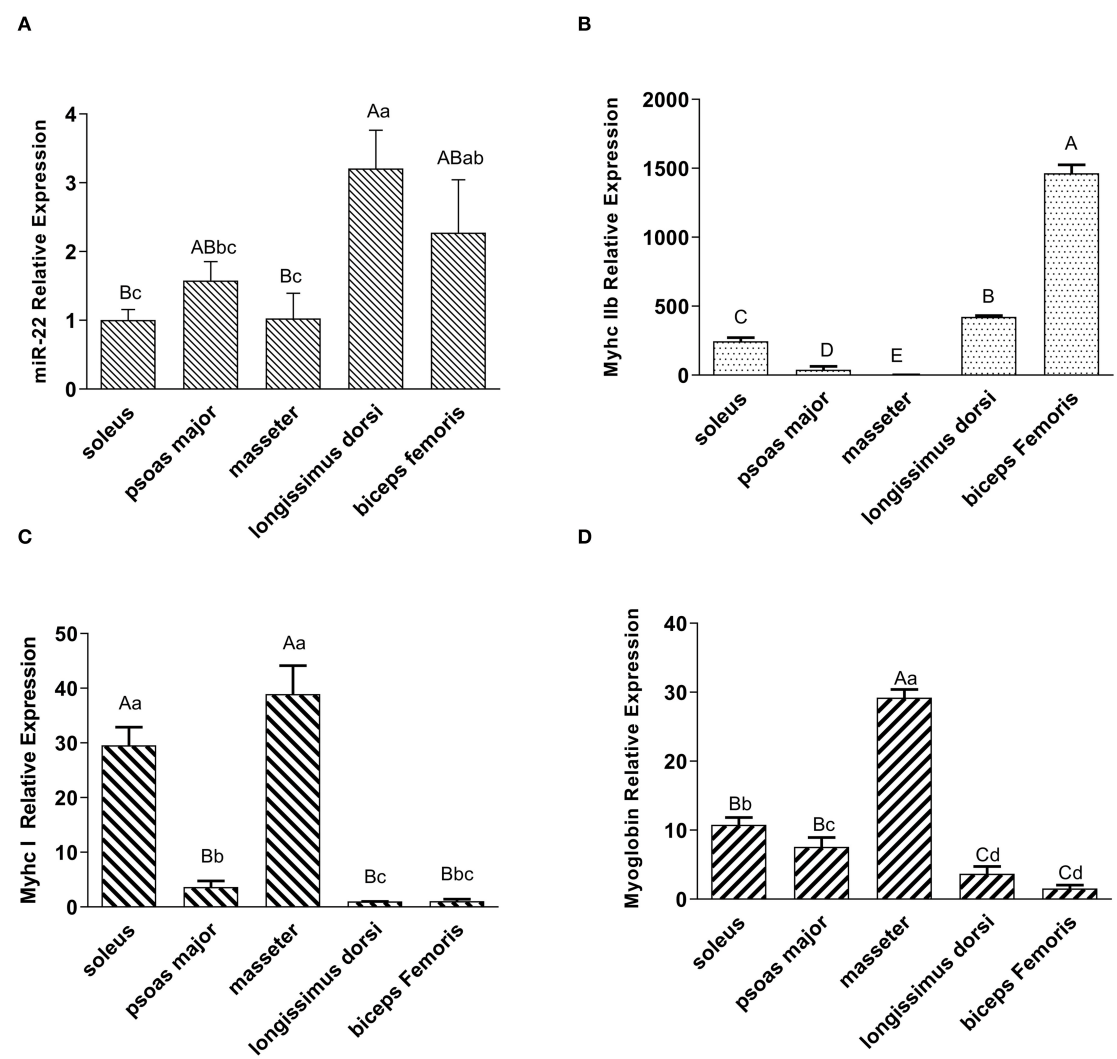
Expression profiling of miR-22, muscle fiber type marker genes, and myoglobin in different types of porcine muscle. **(A)** Expression of miR-22 in different type of skeletal muscles (i.e., SOL, psoas major, masseter, longissimus dorsi, and biceps femoris) in Suhuai pigs. **(B)** Expression of the glycolytic type IIb fiber marker gene *MyhcIIb* in different muscle types. The masseter group was treated as the control group. **(C)** Expression of oxidized type I fiber marker gene *Myhc I* in different muscle types. The longissimus dorsi group was treated as the control group. **(D)** Expression of myoglobin in different types of skeletal muscles. The biceps femoris group was treated as the control group. Capital letters indicate highly significant differences (*P* < 0.01); lowercase letters indicate significant differences (*P* < 0.05) (*n* = 4).

To investigate the expression profile of miR-22 in various muscle types, we performed a qRT-PCR assay. MiR-22 expression was significantly lower in the SOL, PM, and MA muscle than in the LD and BF muscle (*P* < 0.05) (Figure 1A). *Myhc IIb*, a marker gene for fast glycolytic muscle fibers (29), was more highly expressed in the LD and BF muscles than in the SOL, PM, and MA muscles (Figure 1B). However, *Myhc I*, a marker gene for slow oxidative muscle fibers (29), was highly expressed in the SOL and MA muscles (Figure 1C). We also found that *myoglobin* expression patterns were similar to those of *Myhc I* in different muscle types (Figure 1D).

### Descriptive statistics for meat color phenotypes

**Figure 2:**
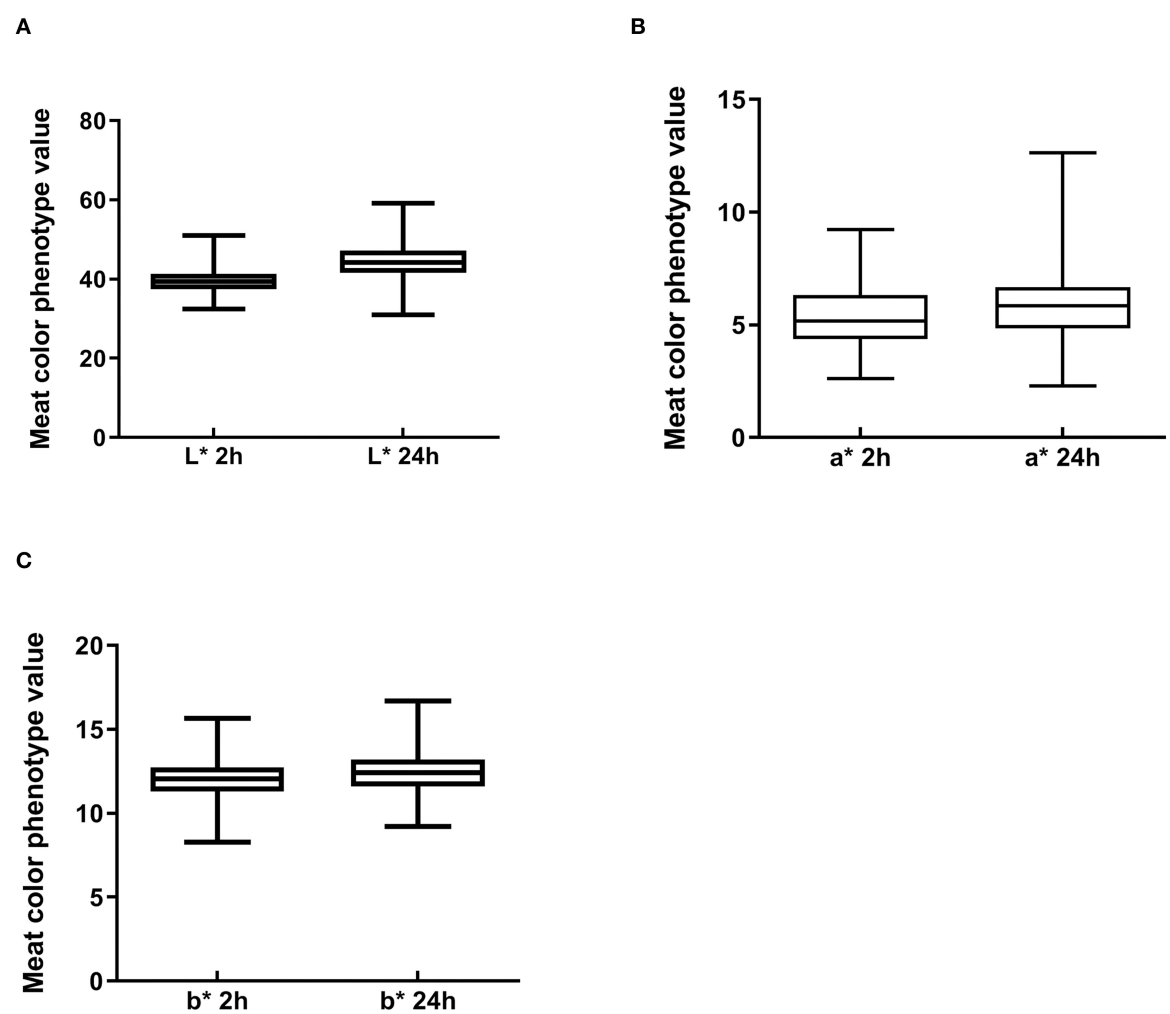
Boxplot of meat color index values in Suhuai pigs at 2 and 24 h after slaughter, including **(A)** lightness (L*), **(B)** redness (a*), and **(C)** yellowness (b*).

To understand the characteristics of the Suhuai pig population, we performed descriptive statistical analysis of meat color trait values. Mean values of meat color traits in this group were within the normal range, although there was a large difference between the maximum and minimum values (Figures 2A–C). The coefficient of variation for meat color a^*^ reached 28.35% at 24 h postmortem (Table 3) and 25.82% at 2 h postmortem (Table 3). The coefficients of variation for all other meat color parameters were <10% (Table 3). These results indicate that the breeding of Suhuai pig meat color should be studied further.

**Table 3:** Descriptive statistics analysis of meat color traits in 300 Suhuai pigs.

**Meat color**	***N***	**Range**	**Mean ± SE**	**CV (%)**	**median**
L* value after slaughter 2 h	300	32.52–51.05	39.54 ± 0.18	7.64%	39.34
a* value after slaughter 2 h	300	2.61–9.22	5.38 ± 0.08	25.82%	5.18
b* value after slaughter 2 h	300	8.28–15.67	12.01 ± 0.06	8.98%	12.04
L* value after slaughter 24 h	300	31.04–59.19	44.43 ± 0.23	8.8%	44.2
a* value after slaughter 24 h	300	2.28–19.18	5.94 ± 0.1	28.35%	5.85
b* value after slaughter 24 h	300	9.22–16.71	12.51 ± 0.07	9.43%	12.41

### Detection of SNP in the miR-22 gene and association analysis of meat color traits

**Figure 3:**
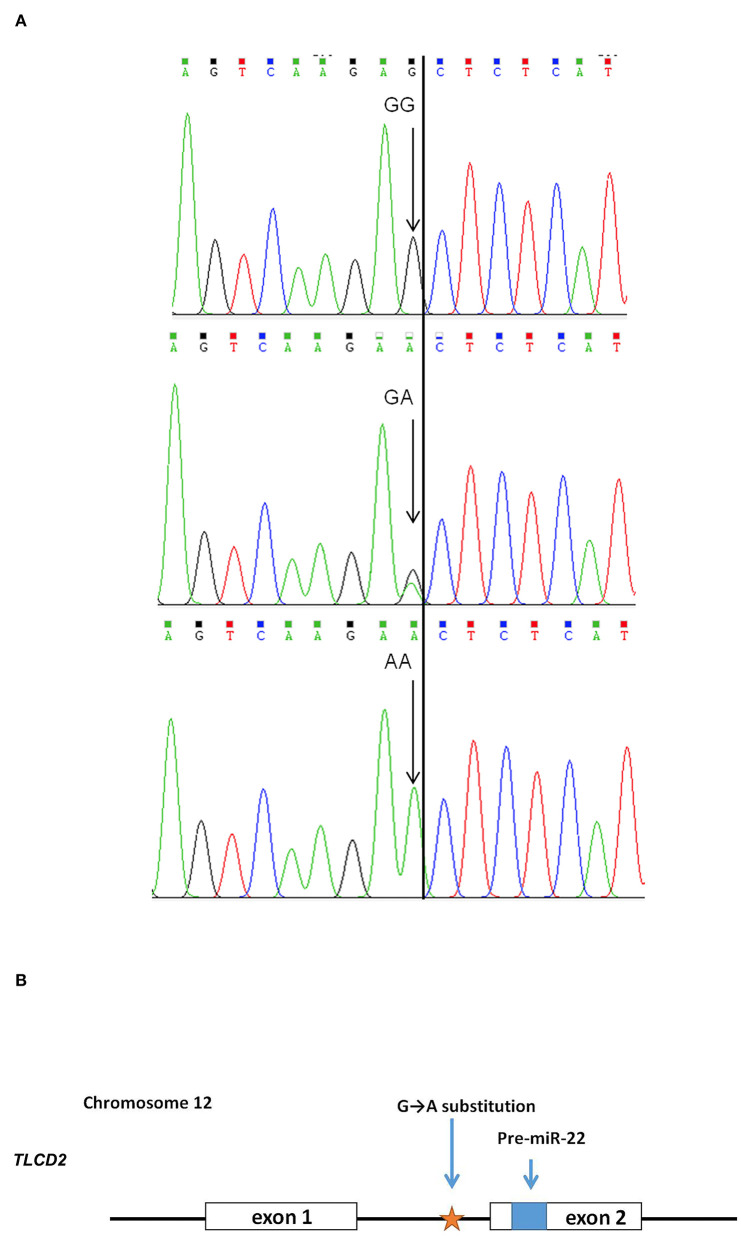
Scheme of the NC_010454.4: g.47913559 G > A mutation in the miR-22 gene. **(A)** Sequencing peak map of the GG, GA, and AA genotypes. Eight individuals were randomly divided into two groups, and each group of four was mixed for sequencing. Arrow indicates the target site. **(B)** Positional relationship between the NC_010454.4: g.47913559 site, ssc-miR-22 precursor, and its host gene *TLCD 2*.

After sequencing the miR-22 genes of eight Suhuai pigs, we found a G/A mutation at NC_010454.4:g.47913559 (Figure 3A) within the 1st intron of the miR22 host gene (*TLCD2*), located 147 bp upstream of pre-miR-22 (Figure 3B). After sequencing the 688 bp DNA amplicons of 300 Suhuai pigs, we found 152, 111, and 37 GG, GA, and AA genotype individuals, respectively (Table 4). The frequency of allele G was 0.735 and that of allele C was 0.265 (Table 4). The NC_010454.4:g.47913559 G > A SNP was significantly associated with meat color a^*^ at 2 h postmortem but not with other meat color parameters (Table 5). At 2 h postmortem, the AA genotype had the highest meat color a^*^ value among all genotypes, and a^*^ was higher for the GA genotype than for the GG genotype (Table 5).

**Table 4:** Allele frequencies of the G > A substitution of miR-22 primary transcript in the Suhuai pig population.

**Population**	**No. of pigs**	**Genotypes**			**Allele frequency**	
Suhuai pig	300	GG	GA	AA	G	A
		152	111	37	0.735	0.265

**Table 5:** Association results for the G > A polymorphism in miR-22 transcript with meat color traits.

**Genotype**	**No**.	Meat color L*at 2 h postmortem	Meat color a*at 2 h postmortem	Meat color b*at 2 h postmortem	Meat color L*at 24 h postmortem	Meat color a*at 24 h postmortem	Meat color b*at 24 h postmortem
GG	152	39.65 ± 0.26	5.08 ± 0.12Bb	11.99 ± 0.09	53.25 ± 7.24	5.83 ± 0.15	12.60 ± 0.10
GA	111	39.46 ± 0.29	5.57 ± 0.13ABa	12.00 ± 0.10	43.66 ± 8.21	6.01 ± 0.17	12.41 ± 0.12
AA	37	38.73 ± 0.50	5.91 ± 0.26Aa	11.91 ± 0.18	43.85 ± 14.02	6.12 ± 0.28	12.37 ± 0.20
*P*-Value		0.2672	0.0008**	0.8933	0.6297	0.5648	0.3659

Capital letters indicate highly significant differences (P < 0.01), and lowercase letters indicate significant differences (P < 0.05).

### Association between miR-22 expression levels and the g.47913559 G > A mutation

**Figure 4:**
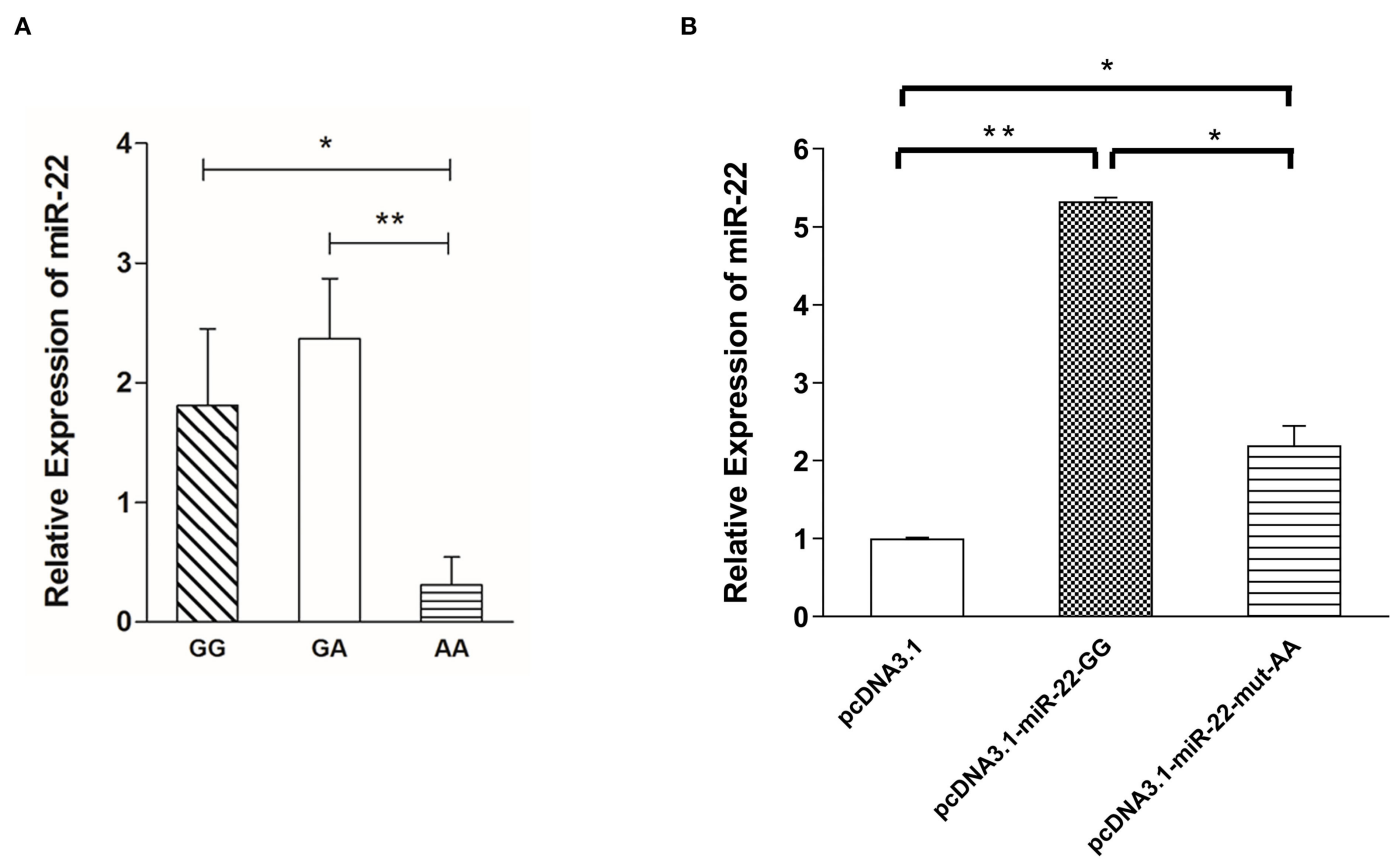
Expression levels of miR-22 are associated with g.47913559 G > A mutation. **(A)** Expression of miR-22 in longissimus dorsi muscle from Suhuai pigs with different genotypes (*n* = 6). **(B)** Expression of miR-22 after transfection of the miR-22 overexpression vector and the mutated vector (*n* = 3). *P < 0.05; **P < 0.01.

To determine the effect of NC_010454.4: g.47913559 G > A on the expression of miR-22 in pig skeletal muscle, we randomly selected six individuals from each genotype to detect the relative expression of miR-22. MiR-22 expression was lowest for the AA genotype, whereas no significant differences were detected between the GG and GA genotypes (Figure 4A). When G > A mutation was performed in the miR-22 overexpression vector, miR-22 expression decreased in the AA genotype compared with the GG genotype (Figure 4B). Together, these results suggest that the NC_010454.4:g.47913559 G > A SNP affects miR-22 expression.

### MiR-22 promotes intracellular Ca^2+^ concentration by targeting *ELOVL6* in porcine skeletal muscle satellite cells

**Figure 5:**
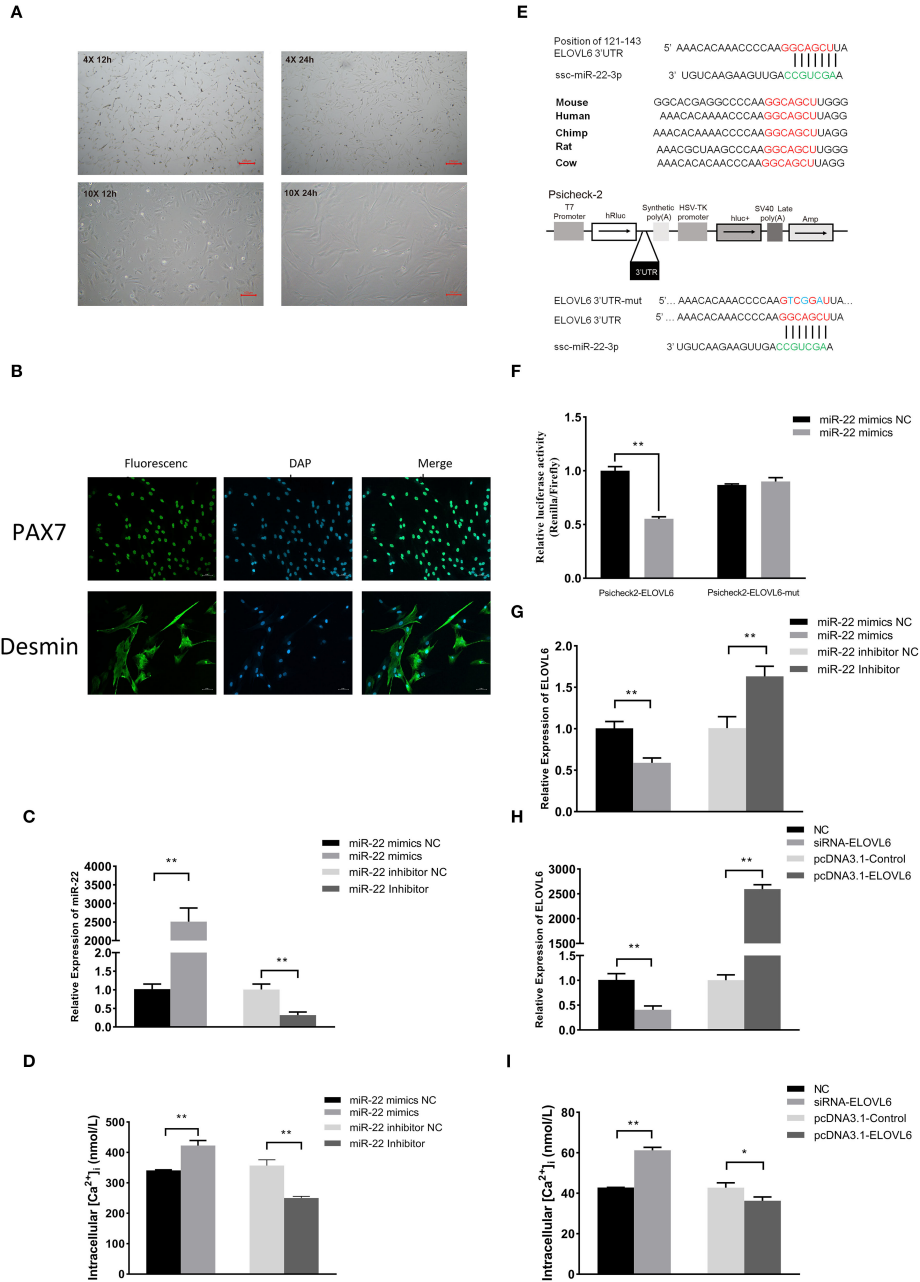
The miR-22 gene promotes intracellular calcium concentration by targeting the elongase of very long chain fatty acids 6 (*ELOVL6*) gene in porcine skeletal muscle satellite cells (PMSCs). Microscopic morphological observation of PMSC growth for 12 and 24 h at **(A)** 4× (upper panel) and 10× (bottom panel) magnification. **(B)** Immunofluorescence staining of satellite cell marker proteins (Pax7 and Desmin) in PMSCs. **(C)** Expression of miR-22 in PMSCs after transfection of miR-22 mimics and its NC and miR-22 inhibitor and its NC. **(D)** Intracellular calcium concentration was measured after PMSCs were transfected with miR-22 mimics, miR-22 mimics NC, miR-22 inhibitor, or miR-22 inhibitor NC for 24 h. **(E)** Sequence of miR-22 and its predicted conserved binding region in *ELOVL6* 3'UTR (red). Structural diagram of the dual-luciferase reporter vector Psicheck-2. The predicted miR-22 target site of the *ELOVL6* 3'UTR and mutated target site were inserted into the 3'-end of the Renilla luciferase gene (hRluc). Firefly luciferase gene (hluc+) expression was used as the standard control. **(F)** HEK293T cells transfected with miR-22 mimics or NC were co-transfected with the Psicheck-2 *ELOVL6* vector or its mutated vector. Relative luciferase activity was determined after 24 h. **(G)** Expression of *ELOVL6* mRNA in PMSCs following transfection of miR-22 mimics, miR-22 mimics NC, miR-22 inhibitor, or miR-22 inhibitor NC. **(H)** Expression of *ELOVL6* mRNA in PMSCs following transfection of siRNA-*ELOVL6*, NC, pcDNA3.1-*ELOVL6*, or pcDNA3.1-Control. **(I)** Intracellular calcium concentration was measured after PMSCs were transfected with siRNA-*ELOVL6*, NC, pcDNA3.1-*ELOVL6*, or pcDNA3.1-Control for 24 h (*n* = 3). *P < 0.05; **P < 0.01.

Isolated PMSCs adhered completely to the cell dish after 12 h of isolation, and the cells were spindle-shaped (Figure 5A). After 24 h, the cells increased in number and grew in a regular manner in one direction. These results indicated that the PMSCs showed vigorous cell viability and normal skeletal muscle satellite cell morphology. To further validate the isolated cells, we performed immunofluorescence staining with the skeletal muscle satellite cell-specific marker proteins *Pax7* and *Desmin* (30). *Pax7* and *Desmin* were positive in the nucleus and cytoplasm, respectively (Figure 5B), further demonstrating that the isolated cells were PMSCs.

Compared with the NC, overexpression of miR-22 effectively increased the intracellular Ca^2+^ concentration, whereas knockdown of miR-22 reduced the intracellular Ca^2+^ concentration of PMSCs (Figures 5C,D). Using the TargetScan online prediction program, we discovered that *ELOVL6* was a potential target of miR-22, with an miR-22 binding site in its 3′UTR region, which is highly conserved in multiple species (Figure 5E). Furthermore, we conducted a dual-luciferase reporter assay to test whether *ELOVL6* is a real target gene of miR-22. The predicted sequence of the *ELOVL6* 3′UTR was inserted into the Renilla luciferase report vector Psicheck-2 (Figure 5E). The mutated Psicheck-2 *ELOVL6* luciferase vector, which has three mutant sites in the binding site of miR-22, was also generated. Overexpression of miR-22 significantly inhibited luciferase activity when co-transfected with Psicheck-2 *ELOVL6* luciferase vector in HEK293T cells (Figure 5F). However, no significant changes in luciferase activity were observed when miR-22 was co-transfected with mutated Psicheck-2 *ELOVL6* luciferase vector.

The expression of *ELOVL6* was significantly downregulated after miR-22 overexpression (Figure 5G) in PMSCs. In contrast, loss of miR-22 upregulated the mRNA expression of *ELOVL6*. SiRNA-mediated knockdown of *ELOVL6* increased the intracellular Ca^2+^ concentration in PMSCs, and overexpressing *ELOVL6* decreased the intracellular Ca^2+^ concentration (Figures 5H,I). These results suggest that miR-22 increases the concentration of intracellular Ca^2+^ by targeting *ELOVL6* in PMSCs.

## Discussion

Genetic and nutritional factors such as breed, muscle fiber type, and feed nutrition, as well as physiological and biochemical factors such as mitochondrial function and lipid oxidation, regulate pig meat color by affecting the content or redox state of myoglobin (31). In this study, we found that miR-22 expression was significantly lower in red muscles with high proportions of oxidative fibers such as porcine SOL, PM, and MA muscles than in white muscles containing high proportions of glycolytic fibers such as the LD and BF. Previous studies have also found that porcine SOL, PM, and MA muscles are mainly composed of oxidized type I and type IIa muscle fibers, whereas muscles such as the LD and BF are mainly glycolytic type IIb muscle fibers (32). Among the reported target genes of miR-22, we discovered genes such as *Sirt1* and *HDAC4*, which promote the formation of red muscles (33, 34). A previous study found that miR-22 eliminated the effects of resveratrol on slow *MyHC* and fast *MyHC* expression in porcine myotubes (35). Our results also showed that *myoglobin* expression was higher in red muscles than in white muscles and that the pattern was opposite to that of miR-22 in different pig muscle types. This finding further suggests that miR-22 expression may be lower in muscles with higher redness values. Therefore, further studies are needed to explore the genes or signaling pathways related to pig meat color regulated by miR-22.

Many studies have analyzed the association between miRNA and economic traits in farm animals through the detection of SNPs in miRNA genes. A T/C mutation in the miR-27a gene, which is associated with litter size in large white pigs, may be a potential molecular marker for litter size trait breeding (36). An SNP on the miR-206 gene is associated with the proportions of type IIa and IIb fibers in muscle and also with meat quality traits including drip loss and backfat and lean meat percentages (37). An SNP on miR-133b is significantly associated with the total number of muscle fibers, loin eye muscle area, and pH (37). In this study, we showed that the coefficient of variation for meat color a^*^ at 2 h postmortem reached 25.82%, such that the meat redness trait has great breeding potential in Suhuai pigs.

Through sequencing, we found a G > A mutation site in the ssc-miR-22 gene that was highly significantly correlated with meat color a^*^ at 2 h postmortem in Suhuai pigs. AA genotype individuals had the highest a^*^value and the lowest miR-22 expression. However, GG genotype individuals had lower a^*^values at 2 h postmortem than GA genotype individuals, but there were no significant differences in miR-22 expression between these genotypes. The G > A mutation in the miR-22 overexpression vector decreased miR-22 expression levels. Thus, this mutation may promote the binding of transcription factors that inhibit miR-22 expression; however, this phenomenon may be fully effective only for AA homozygous genotype. Future studies should further explore the mechanism underlying the effects of this mutation on miR-22 expression.

In a previous study, we found that the *ELOVL6* gene was differentially expressed in different types of muscles in pigs (18), which was in contrast to the expression trend observed in miR-22 in this study. In this study, we also discovered that porcine miR-22 targets *ELOVL6* to regulate the Ca^2+^ concentrations in PMSCs. The calcium/calmodulin kinase II (*CaMK II*) gene is a significantly enriched phosphorylation motif of *ELOVL*6^−/−^ zebrafish compared with wild-type zebrafish (38). In skeletal muscle, *CaMK II* phosphorylation can stimulate intracellular Ca^2+^ levels. Therefore, we speculated that *CaMK II* may be downstream of miR-22 or its target gene *ELOVL6*, where it regulates intracellular Ca^2+^ in PMSCs (39).

In summary, a G/A mutation in the miR-22 gene was discovered to be associated with pig meat color a^*^ at 2 h postmortem, and this mutation influences miR-22 expression. The NC_010454.4: g.47913559 G > A mutation site may act as a molecular marker for meat color in pig breeding. In addition, *ELOVL6* is a direct target of miR-22 in pigs. The effects of miR-22 on skeletal muscle intracellular Ca^2+^ concentrations may be partly due to the inhibition of *ELOVL6* expression.

## Data availability statement

The raw data supporting the conclusions of this article will be made available by the authors, without undue reservation.

## Ethics statement

The animal study was reviewed and approved by the Animal Ethics Committee of Nanjing Agriculture University.

## Author contributions

LH and RH conceived and designed the experiments. HW and ZS performed the experiments. HW, XZ, SY, and JJ analyzed the data. AZ and PL helped to write the manuscript. JJ helped to revise the language. All authors read and approved the final manuscript.

## Funding

This study was supported by the National Natural Science Foundation of China (Nos. 31802030, 32002149, and 32172710).

## Conflict of interest

The authors declare that the research was conducted in the absence of any commercial or financial relationships that could be construed as a potential conflict of interest.

## Publisher's note

All claims expressed in this article are solely those of the authors and do not necessarily represent those of their affiliated organizations, or those of the publisher, the editors and the reviewers. Any product that may be evaluated in this article, or claim that may be made by its manufacturer, is not guaranteed or endorsed by the publisher.
